# Monocular Vision‐Based Endoscopic Sinus Navigation: A SLAM Driven Approach With CT Integration

**DOI:** 10.1049/htl2.70046

**Published:** 2025-12-09

**Authors:** Roger D. Soberanis‐Mukul, Chin Hang Ryan Chan, Ryan Chou, Jan Emily Mangulabnan, Lalithkumar Seenivasan, Xingyu Chen, Mohammad Salehizadeh, S. Swaroop Vedula, Russell H. Taylor, Masaru Ishii, Gregory Hager, Mathias Unberath

**Affiliations:** ^1^ Department of Computer Science Johns Hopkins University Baltimore USA; ^2^ Department of Otolaryngology, Head and Neck Surgery Johns Hopkins Medical Institutions Baltimore USA; ^3^ Department of Cardiothoracic Surgery Stanford University School of Medicine California USA

**Keywords:** computer vision, monocular SLAM, 3D motion estimation, endoscopy, image‐based navigation, sinus navigation, point tracking foundation model

## Abstract

Surgical navigation is critical in sinus surgery to enhance the surgeon's spatial awareness and improve precision, particularly around occluded critical structures. While external tracker‐based navigation systems exist, vision‐based solutions are preferred for being less intrusive and for enabling endoscopic image analysis to assist surgeons. However, monocular endoscopy navigation faces challenges associated with monocular reconstruction and camera pose estimation. This paper presents a proof of concept for monocular vision‐based sinus navigation that utilizes only preoperative CT data and the endoscope video stream to navigate the sinus anatomy. We developed a vision‐based navigation system that incorporates a SLAM algorithm to estimate the camera pose and reconstruct the 3D surface of the anatomy. Given an initial semi‐automated registration, the algorithm maps the SLAM‐based trajectories to the CT space while employing the reconstructed point cloud to solve for the scale interactively. The system displays the updates in the CT triplane visualization as SLAM reconstructs the scene and recovers pose information. We tested our system by performing an off‐site navigation in ten recorded endoscopic video streaming generated from sequences obtained from eight cadaveric subjects, comparing the vision‐based navigation to reference optical tracker pose data and obtaining translation and rotation errors of 3.2 mm and 4.9 degrees, respectively. Additionally, we performed three on‐site tests of our system on two different cadaver experiments. Our work evaluates a fully integrated system that closes the loop between image‐based reconstruction and CT visualization, and discusses the challenges to address to achieve clinical level surgical navigation.

## Graphical abstract

The work presents a vision‐based monocular surgical sinus navigation system. Our method provides the endoscope camera position in the CT space.

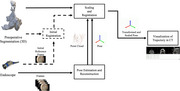

## 1 Introduction

Functional endoscopic sinus surgery (FESS) is a minimally invasive procedure performed to treat chronic sinus conditions such as sinusitis, to remove polyps and other sinus passage blockages, and restore general sinus function and ventilation [1]. FESS uses a thin endoscope to reach the surgical region, reducing the necessity of large external incisions. While this translates to faster recovery times for patients, it also introduces challenges to surgeons who mainly rely on the endoscopic video as the primary source of visual information. Sinus surgeries involve operating near critical structures such as the optical nerve, carotid artery, and the brain [2], making surgical precision and spatial awareness vital for successful surgical outcomes. However, to orient themselves during the procedure, surgeons need to mentally map the location of the endoscope video with a reference anatomical image, like a preoperative computed tomography (CT) scan. This requires a significant mental effort considering the complexity of anatomical structures, limited field‐of‐view, and obscured visibility of occluded structures that restrict surgeon's spatial understanding [3]. Surgical navigation systems aim to address these challenges by providing real‐time, precise guidance information visualized on the preoperative CT. These systems help reduce mental effort and improve spatial awareness, thereby enhancing the ability to operate safely around delicate areas [2, 4]. Conventional surgical navigation systems rely on external tracking technologies that detect and track electromagnetic or visual fiducials affixed to the patient's anatomy and tracking instruments [5]. After an initialization and registration process, these systems provide information about the position relative to the preoperative CT. While these external tracking systems constitute the standard navigation approach, researchers have explored image‐based alternatives. Vision‐based navigation employs primary image information from the endoscope to recover pose and location. Compared with electromagnetic or optical trackers, vision‐based systems have the advantage of not requiring external tracking hardware or markers and are less prone to occlusions or interferences from the surgical team and equipment present in the operating room (OR). Furthermore, the use of images as the primary source of information opens the door to the integration of artificial intelligence (AI) analysis methods into the navigation framework. Such AI models can use the current image information to update the reference CT image as the surgery progresses [6] or provide advanced augmented reality visualization that employs the endoscope location to provide helpful contextual information [7].

Despite the advantages of vision‐based navigation systems, their implementation requires the integration of multiple tasks, including registration, pose estimation, and scene reconstruction, each with its own challenges due to the textureless appearance of the anatomy and the common use of monocular endoscopes. Previous works have contributed to different aspects of this problem. For example, the work presented by Leonard et al. [2] evaluates a system based on structure from motion (SfM). This system employs a sequence of images with SfM to recover the camera pose and the sparse 3D structure of the anatomy. The registration process involves an initial manual alignment of the sparse reconstruction with the CT, refined using the ICP algorithm. The method utilizes electromagnetic tracker information to resolve the scale ambiguity resulting from the use of monocular images. In a different work, Enkaoua et al. [7] present a method that uses a depth sensor affixed to the endoscope to recover pose and structure, while a set of fiducials attached to the external anatomy allows registration. The experiments are performed in a phantom anatomy. The work presented by Bartholomew et al. [8] includes a stereo‐SLAM algorithm for online pose and structure estimation. The method utilizes a stereo endoscope for depth estimation, which feeds the stereo‐SLAM algorithm. Different works presented in the literature address the challenges of a tracker‐free navigation system by relying on different assumptions. Using initial tracking information allows for recovering the scale but still needs to rely on external tracking hardware for initialization. Stereo vision and depth sensors can provide precise structural information but require additional hardware and modifications to the endoscope. Furthermore, monocular endoscopy is the common choice for FESS.

### 1.1 Contributions

This work introduces a tracker‐free, monocular image‐based navigation framework for sinus endoscopy. Our goal is to develop and evaluate a system that utilizes standard operating room equipment, relying solely on a preoperative CT scan and video sequences from a monocular endoscope. Our work introduces a methodology that enables on‐site navigation, where the CT registration process occurs simultaneously as the SLAM model determines the camera's location. We take inspiration from recent advancements in track‐any‐point‐based monocular SLAM [9] that allow out‐of‐the‐box reconstruction, removing the training time requirement of learning‐based approaches [10]. We developed a modular framework that can be updated as new methods arise. We evaluate the system from two perspectives: first, a SLAM retrospective evaluation that compares results after reconstruction is complete, and second, a navigation‐oriented perspective that assesses the system in real‐time as poses are being recovered, with only partial information available at each moment. This latter approach presents a more challenging and realistic scenario. Testing this framework with ten endoscopic videos from cadaver studies, we achieved an in‐navigation error of 3.2 mm in translation and 4.9 degrees in rotation. Additionally, we performed three on‐site tests of the navigation system during two different cadaver experiments, where the surgeon was instructed to align a visual reference on the image with a fiducial inserted in the sinus anatomy.

## 2 Methods

**FIGURE 1:**
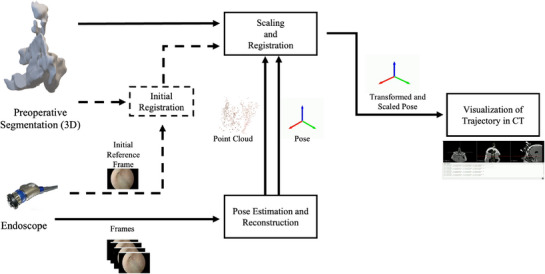
Our sinus monocular endoscopy navigation system consists of three main components: SLAM for camera pose estimation and reconstruction, scaling and registration, and visualization in CT space. The framework utilizes an endoscopic video source along with a CT image and its segmentation to perform a semi‐automated initial registration, which initializes the transformation from the SLAM reconstruction to the CT space. Our navigation system outputs the camera poses in CT coordinates. The estimated camera poses, SLAM‐to‐CT scale, and CT views corresponding to the latest camera poses are iteratively updated and visualized during navigation.

Our navigation system includes three modules for pose estimation, registration and visualization. They are defined to provide guidance and visualization with respect to the preoperative CT image. Figure 1 presents an overall description of the system and the interaction between its different components.

### 2.1 Before Starting: Preoperative Preparation

We reduced the required information into three key elements: an approximate location of the region of interest, a preoperative CT scan of the anatomy, and the endoscopy video stream for navigation. While the third component is specific to navigation runtime, the first two elements are obtained in a preoperative stage. The CT image is acquired and the sinus anatomy is segmented using 3D Slicer [11]. These elements are used for scale recovery and visualization. To define the region of interest, we developed a Python script that visualizes the CT segmentation from a virtual camera's perspective, allowing control of the camera position via keyboard inputs. This process establishes the region of interest and a reference camera pose, $T_{ct}$, within the segmented CT mesh S. Notably, this step does not require specialized hardware and can be performed on a consumer‐level computer.

### 2.2 Initializing the System

**FIGURE 2:**
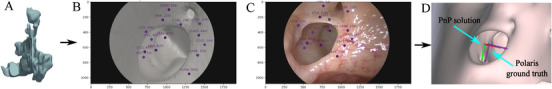
Examples of (A) a segmented mesh model of transnasal cavity. PnP‐based registration process between (B) the CT‐rendered image $I_{ct}$ and (C) the endoscopic view $I_{rgb}$. User‐selected 2D correspondence points were used to compute the initial registration transformation, utilizing the RANSAC PnP solver [13]. (D) Initial transformation frames from Polaris as ground truth and from PnP solution shown across sinus mesh in Meshlab environment. The optical tracker poses shown is only for reference.

Like many navigation systems, our pipeline requires an initial registration to establish the transformation between recovered camera poses and the CT coordinate frame. For this study, we assume that we will navigate a specific region of interest within the sinus anatomy, and navigation begins once we reach a predefined starting point. We first use the CT segmentation and the reference pose $T_{ct}$ (obtained in the preoperative stage) to render a reference image $I_{ct}$ and depth map $D_{ct}$. This process is expressed as: $\left(I_{ct},D_{ct}\right)=\text{render}\left(S,T_{ct}\right)$. Assuming the endoscope is positioned around the starting region, the first endoscopic frame image ($I_{rgb}$) of the navigation system's SLAM backbone is registered to the CT space using the reference image $I_{ct}$. To register the two frames, a set of 2D correspondences $x_{ct}\in I_{ct}$ and $x_{rgb}\in I_{rgb}$ is manually defined between the frames (Figure 2). This part of the process is analogous to the pivot‐based initialization used in standard navigation systems. Using the reference pose $T_{ct}$, depth map $D_{ct}$ and the endoscope calibration matrix K, the 2D point correspondences ($x_{ct}$, $x_{rgb}$), are projected into 3D points using the equation: $x_{rgb}=KT_{rgb}X_{ct}$. We then employ perspective‐n‐point (PnP) [12] between these 3D points correspondences to obtain the pose of the first endoscopic frame $I_{rgb}$ with respect to the CT coordinate frame. Finally, this pose is used to establish the registration between the reconstruction and CT image.

### 2.3 Monocular Vision‐Based Navigation Modules

Once the system is initialized, the navigation process continuously iterates through three steps: pose estimation, scaling and registration, and visualization in the CT reference image, as described below.

#### 2.3.1 Pose Estimation Module

The pose estimation module incorporates OneSLAM [9] as the monocular SLAM backbone to recover pose and sparse structure. Leveraging CoTracker [14], a robust point tracking foundation model, and local bundle adjustment, OneSLAM demonstrated comparable performance to existing domain‐specific SLAM approaches in generating sparse point cloud of the anatomy and estimating the endoscope camera trajectory. OneSLAM's CoTracker model tracks 2D points based on a sliding window approach, where the correspondences are computed for a set of n=8 frames at a time, with a step size of 4 frames. To accumulate and filter point correspondences predicted by the CoTracker for the sequence of images $\left\{I_{T-l_{m}},\text{…},I_{T}\right\}$, OneSLAM maintained a section buffer of length $l_{m}$ where m>n. This window advances with a step size of $l_{m}-1$ and frames are accumulated until the section buffer is refilled. Then, it runs a local bundle adjustment process on the accumulated section to estimate the pose and the sparse structure. Once OneSLAM computes the next keyframe pose and sparse reconstruction, the registration module process this information to close the loop between the image‐base reconstruction and the CT reference image.

#### 2.3.2 Continuous Scaling and Registration Module

The ambiguity in monocular reconstruction creates an unknown scaling factor between the SLAM‐based reconstruction and the preoperative CT. We compute this scaling factor using 3D point correspondences between the preoperative CT segmentation and the SLAM‐reconstructed point cloud. To find an initial scaling factor, we use the point cloud obtained after the second keyframe in SLAM. From this, we extract 3D points visible from the first keyframe and their corresponding 2D image positions. Using the transformation from the first keyframe described in Section 2.2, we trace rays from the 2D image points to the CT surface using Open3D. This establishes correspondences between the CT and the reconstructed anatomy, preserving their relative proportions despite differing scales. Finally, we calculate the scaling factor by determining the ratio of the lengths of the longest lines defined in each point cloud. This method provides an initial scaling factor. However, SLAM‐based algorithms improve their accuracy as more poses are observed. During initial reconstruction, uncertainty about the 3D points can affect the calculated scale, leading to drift and reduced registration accuracy. To tackle this issue, we use a continuously updating scaling factor that adjusts as more frames are observed and 3D point density increases, thereby reducing uncertainty. Each time OneSLAM adds a new keyframe, the scale is updated using all reconstructed points visible from the first camera. This process is managed by the registration module, which also applies the PnP‐based transformation to align the predicted camera poses with the CT coordinate system. Once the initial registration and scaling factor are defined, the main operative loop performs an iteration of OneSLAM and applies the scale and transformation to the predicted pose, which is then sent to visualization modules to display the information in the preoperative CT reference image.

### 2.4 Visualization Module

**FIGURE 3:**
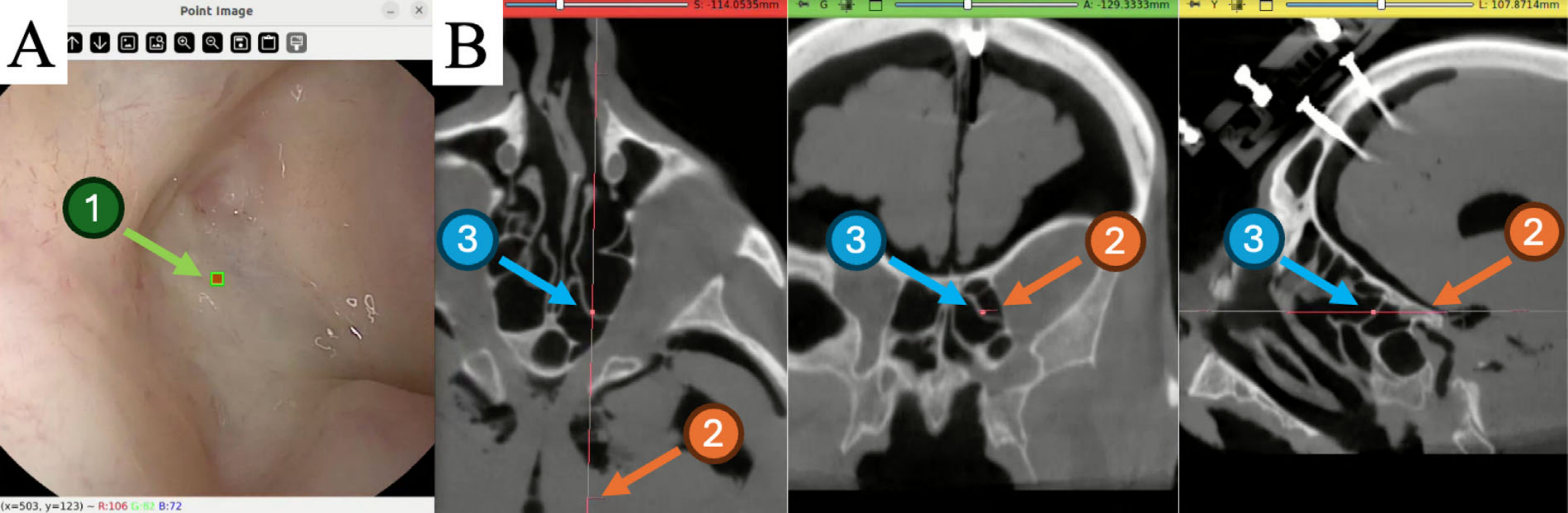
(A) Image endoscope reference view and (B) CT slices of the explored anatomy. In this proof of concept, (1) shows a visual reference in the image that correlates to the CT visual marks. Using the estimated camera pose, we project a virtual line in CT space through the reference (1). This line is indicated by (2). Finally, (3) represents the intersection of the projected line (2) with the tissue. The TRI‐plane view is center at this intersection point and updates its position as the endoscope moves.

The visualization module uses the registered camera pose to provide surgeons with guidance through three views of the preoperative CT image, primarily utilizing 3D slicer for visualization. It displays a perpendicular line and its point of intersection with anatomical tissue and updates views in the sagittal, coronal, and axial planes as the camera moves. This setup provides location and trajectory information of the rigid endoscope tube, resembling the pivot pointer in traditional tracker‐based navigation systems. Figure 3 shows a view of the estimated endoscope position and point of intersection in the CT image.

## 3 Experiments and Results

### 3.1 Dataset and Implementation

We evaluated the navigation system using an in‐house dataset from ten nostrils of eight human cadaveric subjects. An experienced surgeon performed a preoperative inspection of the sinus cavity, focusing on the inferior and middle turbinate. Although the study involved ex‐vivo specimens and does not meet the criteria for human subjects research, it was ethically reviewed and approved under the approved protocol IRB00267324 on non‐living human subjects, for which informed consent or ethics agreement was not required. The dataset includes CT images from a Brainlab LoopX scanner (Brainlab, Munich, Germany) and synchronized endoscopic videos from a Storz Image1 HD camera (Karl Storz SE & Co. KG, Tuttlingen, Germany), with camera poses recorded by an NDI Polaris Hybrid Position Sensor (Northern Digital Inc., Waterloo, Canada). The average number of frame per sequence is 431 with a minimum of 56 frames and a maximum of 788.

The system was implemented, and all our experiments were conducted on a consumer‐level workstation equipped with a single NVIDIA GeForce RTX 4090 graphics card with 24 GB of VRAM, alongside an Intel i9 processor and 32 GB of RAM.

### 3.2 Evaluation Metrics

We evaluate the recovered poses with respect to the optical tracker measurements. Each component(rotation and translation) of the pose is evaluated independently following similar error metrics as Hernandez et al. [15]. The translation error is given by the L2 distance between the translation component of the optical tracker poses and the estimated poses. The rotation is evaluated considering the residual rotation between the optical tracker and pose estimation principal rotation axes. This residual is computed as $R_{\Delta }=R\cdot R_{tracker}^{-1}$. Then, the final error metric is obtained from this residual as [15, 16]:

(1) $$∥\theta_{\Delta }∥=\arccos\left(\frac{\text{tr}(R_{\Delta })-1}{2}\right)$$

We also provide an analysis of the individual x, y, and z components of the rotation error. This error is calculated as the angle between the corresponding axes of the estimated pose and the optical tracker pose. For instance, we measure the angle between the *x*‐axes of the optical tracker and the estimated pose when the translations of both poses are aligned.

### 3.3 Off‐Site Navigation

The off‐site navigation evaluation utilizes a CT image of the anatomy and its associated recorded endoscopic video as inputs, requiring no additional information for navigation. Although this experiment utilizes a recorded video, live endoscopic streams can be used as an alternative to video recording. We assessed the errors generated by the SLAM algorithm alone, as well as the navigation error of the integrated navigation system, which incorporates all its modules.

The SLAM errors provide estimates of translation and rotation errors after the SLAM algorithm has processed the entire sequence. The resulting trajectory is then compared to the trajectory obtained from an optical tracker, establishing a baseline for the system when all necessary elements for scale recovery and registration transformation are available. This analysis is similar to the retrospective evaluation discussed in previous studies [2, 8], where information from electromagnetic trackers or fiducials is utilized to recover scale and register the obtained poses. In our case, we run an independent SLAM process on each sequence, and then we use the information from the optical tracker to recover the scale and registration transformation.

In contrast, the navigation error estimates the translation and rotation errors when the modules described in the method section recover the scale and image‐to‐CT transformation as the SLAM model reconstructs the scene. For this analysis, no retrospective registration is required, and the recovered poses are directly compared with the optical tracker measurements. Although a pre‐recorded video is used, the system registers the poses to the CT space as they are recovered, creating a scenario similar to a prospective evaluation. This presents a more challenging case, as only partial information is available to determine the scale and registration. We assume that no fiducial markers or previous tracker information are available for recovering these values. By comparing the retrospective SLAM errors with the in‐navigation errors, we gain insights into the system's performance and the impact that the modules have on it. Since SfM is a well‐established reconstruction method used when all frames are available for reconstruction, it was included as a reference baseline, using the COLMAP implementation [17, 18]. Previous studies have also utilized SfM for retrospective navigation [2]. However, this approach has the drawback of needing all frames to be accessible, which limits its use for real‐time on‐site navigation. Consequently, only the post‐reconstruction registration errors are reported for SfM, since navigation errors (pose estimation for continuously incoming frames) can not be obtained.

**FIGURE 4:**
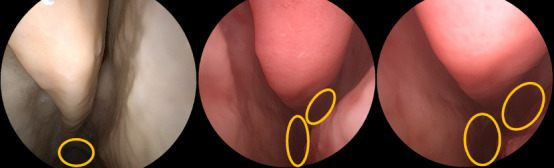
Off‐site evaluation data image examples shown. Some of the errors in translation can be explained by scaling factor issues due to large depths, where shadows are generated when the sinus wall is far from the camera, and to noise in the image. Major depth range shadows are circled.

Results are presented in Table 1. For SfM, it is worth noting that the model was unable to converge or estimate the pose of more than 50% of the cameras in three of our sequences. The number reported for SfM does not include these unsuccessful reconstructions. This can be attributed to the limitations of the keypoint descriptor commonly used with SfM and the challenging texture of the anatomical tissue [19]. However, because SfM performs a global optimization across all frames, it produces the best balanced performance in terms of translation and rotation and serves as an upper benchmark for SLAM‐based methods. The retrospective analysis of the monocular SLAM algorithm alone shows that it can recover camera poses with an average error of 1.4 mm and a general rotation error of 2.7 degrees. When the registration and scaling are computed as SLAM recovers the structure — in‐navigation error — errors increase by 1.8 mm for translation and 2.2 degrees for rotation. While some of the rotational navigation errors can be attributed to the initialization of the registration and the quality of CT segmentation, we found that certain differences in the translation errors arise from challenging scenarios for SLAM. Specifically, issues such as large distances, the shadows they create, and noise in the images contribute to outliers in point cloud estimation, which in turn affects the in‐navigation scale estimation, and hence the translation when registered to the CT space (Figure 4).

**TABLE 1:** Translation (mm) and rotation (degrees) average errors and standard deviation for the selected SLAM backbone (OneSLAM), and the navigation system. A comparison of the performance with a different backbone (SAGE SLAM [10]) and an ablation on the scaling factor computation are also included.

Method	Translation (mm)	General rot (deg)	Rot X (deg)	Rot Y (deg)	Rot Z (deg)
SfM reference baseline	1.6 ± 1.4	1.7 ± 2.2	1.0 ± 1.5	0.6 ± 0.4	1.1 ± 1.5
SAGE backbone SLAM only	2.2 ± 1.5	5.8 ± 2.5	2.3 ± 1.1	3.4 ± 1.6	3.8 ± 1.8
SAGE backbone navigation	4.4 ± 2.3	7.4 ± 2.8	3.8 ± 1.6	4.4 ± 1.7	4.2 ± 1.8
OneSLAM backbone SLAM only	1.4 ± 0.8	2.7 ± 2.5	1.4 ± 1.4	1.2 ± 1.3	1.7 ± 1.5
OneSLAM backbone navigation	3.2 ± 1.4	4.9 ± 2.6	2.8 ± 1.6	2.5 ± 1.5	2.8 ± 1.7
OneSLAM backbone navigation no cont. scaling	5.6 ± 3.3	4.9 ± 2.6	2.9 ± 1.5	2.5 ± 1.5	2.7 ± 1.6

While OneSLAM [9] is our main SLAM backbone, we also perform a comparison of the system with SAGE [10] as its SLAM backbone. This experiment also demonstrates the modularity of our framework, which allows the use of different SLAM models. SAGE is a learning‐based SLAM algorithm proposed for sinus endoscopy. Results are presented in Table 1. SAGE presents comparable performance to OneSLAM, with a difference of 1 mm in translational error. Differences in rotation are in the range of two degrees. Overall, OneSLAM outperforms SAGE on the tested sequences. Additionally, SAGE exhibits similar increases in navigation error, indicating that the challenges discussed earlier can impact the performance of different SLAM methods. Based on these results, we performed the remaining experiments using OneSLAM as backbone.

We also evaluate the effect of the continuous scaling factor incorporated in the registration module. We set a constant scale of one — no continuous scaling — and compute the navigation errors. The results presented in Table 1 indicate that the error for translation increases by 4.2 mm compared to the retrospective SLAM error. The error for rotation remains the same as the version with continuous scaling, as scale primarily influences the camera's position. The increase in translation error highlights the advantages of our continuous scaling estimation integrated into the navigation system.

We finally assessed the consistency of the point cloud generated by the SLAM backbone in relation to the CT structure. To accomplish this, we utilized the 3D‐2D correspondences estimated by SLAM and the camera poses obtained from the optical tracker to establish a set of 3D correspondences between the CT segmentation and the recovered point cloud. This process involved projecting the 2D coordinates of the SLAM keypoints onto the CT using the optical tracker poses. For each patient, we used half of the SLAM point cloud to compute the registration and the other half to calculate the error, simulating a target registration error (TRE) evaluation. The results are presented in Table 2. The residual represents the average squared distance between the correspondences used for registration, while the TRE indicates the error between the remaining corresponding points. Both errors fall within a three‐millimeter range, which is consistent with and lower than the expected six‐millimeter errors range reported for the selected SLAM backbone [9].

**TABLE 2:** Average ± standard deviation of the point cloud obtained by the SLAM backbone compared with the CT segmentation.

Residual error (mm)	TRE (mm)
3.3 ± 0.8	3.5 ± 0.8

### 3.4 On‐Site Navigation

We conducted a preliminary on‐site study involving three endoscopic sinus inspections on two cadaveric subjects. In this on‐site experiment, the video stream originates from an endoscope manipulated by the surgeon. The endoscopic video signal was streamed to the main computer using the robotic operating system (ROS). Unlike our off‐site experiments, this test was carried out in the postoperative stage of surgery, following a simulated ethmoidectomy procedure on the cadaver. Before starting the experiment, we obtained a CT scan and performed the segmentation of the sinus cavity. Additionally, we calibrated the endoscopic camera. For the on‐site experiment, we integrated an image undistortion stage into the OneSLAM data loader. Optical tracker information was also recorded at the same time for comparison purposes.

**FIGURE 5:**
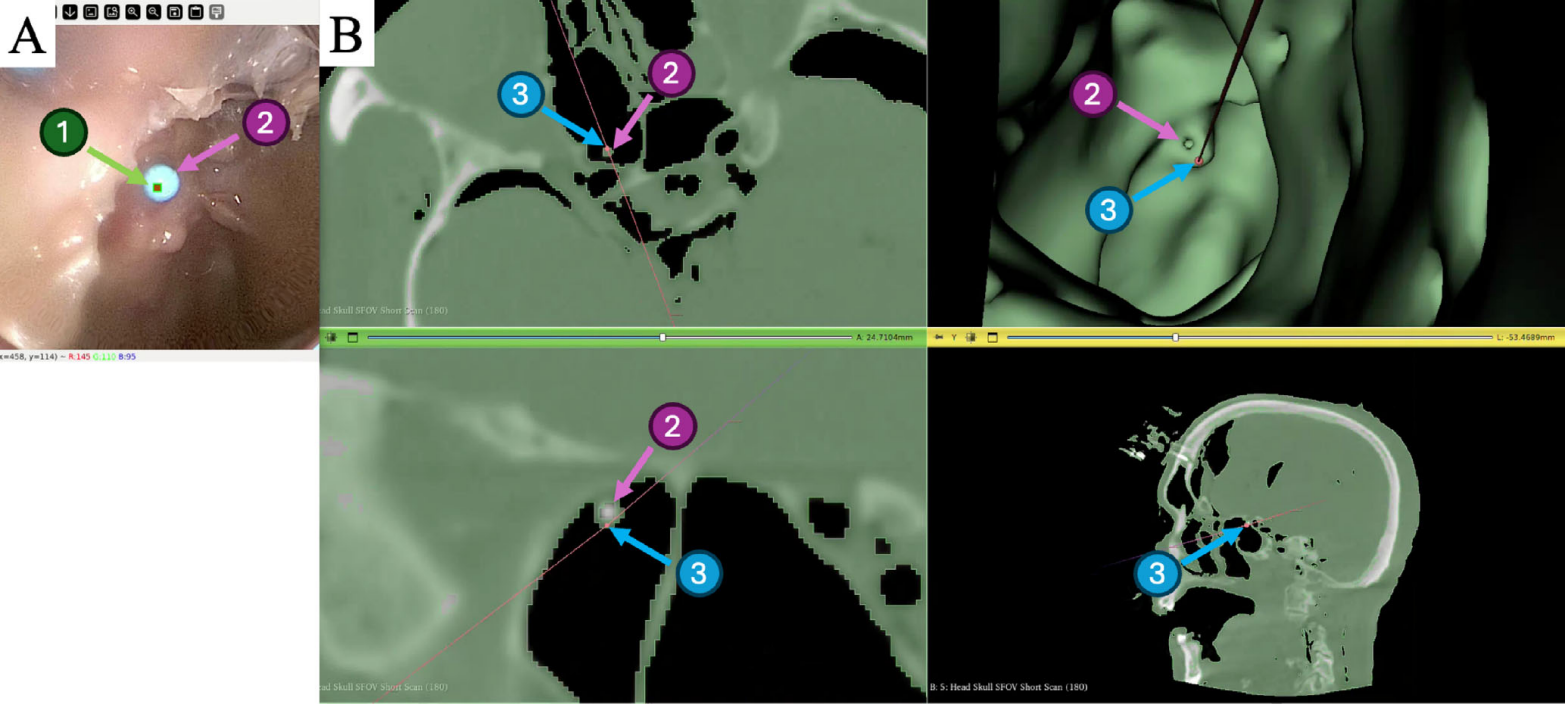
(A) Image endoscope reference view, (B) axial, coronal and sagittal views of the explored anatomy, and a view of the CT segmentation. (1) shows a visual reference in the image, centered on a fiducial marker (2). (2) is also highlighted in the CT scan. (3) represents the projection of the visual reference (1) onto the CT scan.

During the test, we requested the surgeon to align the central image reference (label (1) in Figure 5) with a CT‐visible (fiducial) marker, while system displayed the corresponding projection in the CT image in 3D Slicer. We evaluated the accuracy of the estimated camera projection using this fiducial marker placed within the anatomical structure. This marker has a diameter of 2.3 mm. This process was repeated one time in the first cadaveric subject, and two times in the second subject. After the experiment, we segmented the fiducial and computed the point‐to‐mesh distance between the estimated points of intersection and the segmented marker in the CT space. Figure 5 illustrates a view of the experiment, where the fiducial marker is visible in both the endoscopic image and the CT image.

Table 3 reports the distance between the estimated projection and the segmented fiducial marker, provided the image reference point aligns with the marker (see Figure 5A). It also outlines the translation and rotation errors of the estimated poses in relation to the optical tracker estimations. When the image reference point intersects the fiducial marker, the average distance to the marker across the three inspections is 1.3 mm, indicating a low error in the estimated projection. The average translation error is approximately 4.5 mm and the rotation around the camera main rotation axis is 9.9 degrees.

**TABLE 3:** Prospective errors during the on‐site experiment. We report the point‐to‐mesh distance between the estimated projection and the segmentation of the fiducial marker positions. We also report the navigation translation and rotation errors when comparing with the optical tracker. The results include the general and per‐axis (*x*, *y*, *z*) errors.

Distance to Marker (mm)	Transl. (mm)	Transl. *x* (mm)	Transl. *y* (mm)	Transl. *z* (mm)	Rot. (deg)	Rot. *x* (deg)	Rot. *y* (deg)	Rot. *z* (deg)
1.3 ± 0.6	4.5 ± 2.8	1.6 ± 1.1	2.9 ± 3.0	1.6 ± 0.7	9.9 ± 3.0	6.3 ± 2.2	5.4 ± 2.0	5.0 ± 1.4

We noticed and increment in the estimated error compared with the average obtained during off‐site navigation. These differences may arise from the complex movements of the surgeon when aligning both references, which can introduce errors in the reconstruction process. These errors could affect the scale computation compared to the simpler scoping performed during the sequences evaluated in the off‐site analysis. When we examined the individual components of the translation error, we found that the *y*‐axis contributes the most to the total error, with a value close to 3.0 mm for this axis, while the errors from the other camera axis are within the 2.0 mm range. Overall, a possible reason is that the computed scale may either underestimate or overestimate the position, leading to the observed differences in pose.

In terms of rotation, the individual rotation deviations along the *x*, *y*, and *z* axes, as shown in Table 3, indicate that the *x* and *y* axes contribute approximately five to six degrees to the overall rotation. In contrast, the differences in angles between the *z*‐axes of the optical tracker poses and the estimated poses fall within a range of five to degrees. The *z*‐axis aligns with the camera's principal point. These results suggest that the estimated pose maintains a low orientation error along the principal axes, while the rotation primarily occurs around the *x* and *y* axes within the camera plane. Additionally, while the small distance to the CT fiducial marker indicates the effects and contribution of these measured errors in the final projection, an extended analysis is necessary to better understand the sources of these errors and their impact during on‐site navigation.

### 3.5 Limitations and Future Work

Monocular reconstruction of anatomical tissue faces several challenges, including reconstructing anatomy from textureless images and dealing with scale ambiguity inherent in monocular vision. These limitations can impact the registration to CT images, which is essential for effective surgical guidance. Our navigation system aims to bridge the gap between the reconstruction process and CT‐based visualization. While our contributions advance this objective, we acknowledge the current limitations and challenges that will guide our future work. One limitation of our current approach is the reliance on PnP‐based initialization, which requires user input. Future research aims to minimize user interaction during this initialization process by implementing an automatic image‐based method for RGB‐to‐CT camera localization within the anatomy. Furthermore, such registration methods can contribute to improved camera pose estimation by providing feedback that the SLAM backbone can use for pose correction. Another area for improvement is the robustness to camera reinsertions. As the surgery progresses, the endoscope may leave and re‐enter the anatomy, making camera relocalization a critical challenge for navigation. Camera relocalization can be defined as the problem of recovering the pose and registration when the endoscope leaves and re‐enters the anatomy. Additionally, our off‐site and on‐site experiments revealed that while we can recover an estimation of the reconstruction scale, this estimation depends on the quality of the point cloud generated by the SLAM algorithm. Future work will focus on developing efficient methods to recover the missing scale. Additionally, there is potential for improvement in monocular SLAM algorithms to reduce ambiguity during scale computation. In our off‐site experiments, the system operates at 10 frames per second. However, communication with the endoscope during on‐site experiments reduced this frame rate to three frames per second. A higher frame rate can also benefit the performance of the SLAM model by allowing more frames to be processed during on‐site navigation. Time performance gains can often be achieved through technical improvements, such as optimizing communication and reducing code runtime. While the surgeon successfully utilized the system to align the references, focusing on further optimizations of the algorithmic and communication components is fundamental for future enhancements to the pipeline.

## 4 Conclusion

In this work, we presented a proof of concept for a monocular vision‐based navigation system that incorporates CT registration in the process. We conducted a retrospective evaluation of the SLAM backbone and a prospective analysis of the navigation system as it reconstructed both pre‐recorded and live video streams. Our work evaluates the feasibility of developing a monocular vision‐based navigation system integrated with CT preoperative images and discusses its current limitations, which define future steps toward clinical implementation.

## Author Contributions

Roger D. Soberanis‐Mukul and Chin Hang Ryan Chan share first authorship and contributed to the coding, experimental design and execution, data collection, and writing the manuscript. Ryan Chou and Mohammad Salehizadeh contributed to the code and revision of the manuscript. Jan Emily Mangulabnan contributed to data collection and preprocessing. Lalithkumar Seenivasan and Xingyu Chen contributed to revisions of the SLAM process. S. Swaroop Vedula and Masaru Ishii contributed to the organization and implementation of the ex vivo studies, endoscopic inspection, and clinical mentorship. Russell H. Taylor, Gregory Hager, and Mathias Unberath are the technical mentors, and contributed to the definition of the methodology, and revising the manuscript.

## Funding

This work was supported in part by NIH R01EB030511 and by Johns Hopkins University internal funds. Mohammad Salehizadeh was supported in part by Canadian Network for Research and Innovation in Machining Technology, Natural Sciences and Engineering Research Council of Canada (Grant No. PDF‐568427).

## Conflicts of Interest

The authors declare no conflicts of interest.

## Data Availability

Data is not publicly available.
